# Herpesvirus infections in adenoids in patients with chronic adenotonsillar disease

**DOI:** 10.1002/jmv.27818

**Published:** 2022-05-11

**Authors:** Lotta E. Ivaska, Antti Silvoniemi, Emilia Mikola, Tuomo Puhakka, Matti Waris, Tytti Vuorinen, Tuomas Jartti

**Affiliations:** ^1^ Department of Otorhinolaryngology—Head and Neck Surgery, Turku University Hospital University of Turku Turku Finland; ^2^ Department of Otorhinolaryngology Satakunta Central Hospital Pori Finland; ^3^ Institute of Biomedicine, Division of Infections and Immunity University of Turku Turku Finland; ^4^ Department of Clinical Microbiology Turku University Hospital Turku Finland; ^5^ Department of Pediatrics and Adolescent Medicine, Turku University Hospital University of Turku Turku Finland; ^6^ PEDEGO Research Unit, Medical Research Center University of Oulu Oulu Finland; ^7^ Department of Pediatrics and Adolescent Medicine Oulu University Hospital Oulu Finland

**Keywords:** adenoid, Epstein–Barr virus, human herpesvirus 6, human herpesvirus 7, nasopharyngeal aspirate, palatine tonsil, respiratory viruses

## Abstract

Adenoids and tonsils have gained interest as a new in vivo model to study local immune functions and virus reservoirs. Especially herpesviruses are interesting because their prevalence and persistence in local lymphoid tissue are incompletely known. Our aim was to study herpesvirus and common respiratory virus infections in nonacutely ill adenotonsillar surgery patients. Adenoid and/or palatine tonsil tissue and nasopharyngeal aspirate (NPA) samples were collected from elective adenoidectomy (*n* = 45) and adenotonsillectomy (*n* = 44) patients (median age: 5, range: 1–20). Real‐time polymerase chain reaction was used to detect 22 distinct viruses from collected samples. The overall prevalence of herpesviruses was 89% and respiratory viruses 94%. Human herpesviruses 6 (HHV6), 7 (HHV7), and Epstein–Barr virus (EBV) were found, respectively, in adenoids (33%, 26%, 25%), tonsils (45%, 52%, 23%), and NPA (46%, 38%, 25%). Copy numbers of the HHV6 and HHV7 genome were significantly higher in tonsils than in adenoids. Patients with intra‐adenoid HHV6 were younger than those without. Detection rates of EBV and HHV7 showed agreement between corresponding sample types. This study shows that adenoid and tonsil tissues commonly harbor human herpes‐ and respiratory viruses, and it shows the differences in virus findings between sample types.

## 1 INTRODUCTION

Adenoids and palatine tonsils (in this article tonsils) are secondary lymphoid tissue and serve as a new in vivo model to investigate virus persistence, latent virus infections, and immune functions.^1^, ^2^, ^3^ It is well known that respiratory virus infections are commonly prevalent in tonsils, adenoid, or nasopharyngeal aspirate (NPA) of patients undergoing adenotonsillar surgery.^1^, ^4^, ^5^ However, different viruses are not equally distributed between adenoids and tonsils. For example, human bocavirus 1 (HBoV1) seems to have tropism in adenoid tissue (25%).^6^ In the recently published SPLIT study, human herpesviruses 6 (HHV6), 7 (HHV7), and Epstein–Barr virus (EBV) were the most common herpesviruses found in the tonsil tissue (50%, 70%, 70%).^7^ Furthermore, HHV6, HHV7, EBV, and cytomegalovirus (CMV) have been detected in adenoid tissue of elective adenotonsillectomy patients, the prevalence rates fluctuating in the ranges of 20%–77%, 50%–77%, 43%–73%, and 3%–50%, respectively.^8^, ^9^, ^10^

Ubiquitous human herpesvirus infections are usually acquired via droplets of respiratory tract secretions or saliva during childhood or adolescence. The primary infections are generally asymptomatic or induce mild symptoms. Herpesviruses escape the host's immune system and persist for life. HHVs can cause severe infections and predispose immunocompromised hosts to end‐organ disease and lymphomas.^7^, ^11^, ^12^ Furthermore, EBV is known to associate with nasopharyngeal carcinoma and with certain lymphomas.^13^, ^14^ In general, it is estimated that virus infections are associated with up to 10% of newly diagnosed cancers annually.^15^ CMV, HHV6, and HHV7 are known to persist in T‐cells, whereas EBV persists in B‐cells. HSV1, HSV2, and VZV are neurotropic.^7^, ^8^, ^9^, ^10^ Still, the persistence and distribution of herpesviruses in secondary lymphoid tissue remain unclear. Particularly the role of adenoid tissue is not completely known.

There are several studies concerning respiratory viruses of tonsil and adenoid tissue or sole herpesviruses of tonsil and adenoid tissue. However, there are limited data of persistent herpesvirus and respiratory virus infections in autologous adenoid, tonsil, and NPA samples.^9^, ^16^ Since herpesviruses are a burden particularly to immunosuppressive patients, causing reactivation, and EBV has oncogenic capacity, it is essential to study virus reservoir and tissue tropism. The aim of this cross‐sectional study was to analyze the presence of seven human herpesviruses: herpes simplex viruses 1–2 (HSV1, HSV2), varicella‐zoster virus (VZV), EBV, CMV, HHV6, and HHV7 in adenoid and tonsil tissues and NPA. Common respiratory viruses were also detected from adenoid, tonsils, and NPA samples. Additionally, we compared the difference in virus findings between adenoid and tonsil tissues. We hypothesized that a high level of human herpesvirus genomes is found in secondary lymphoid tissue.

## 2 PATIENTS AND METHODS

### 2.1 Study patients

2.1.1

NPA samples, adenoid, and/or tonsil tissue samples were collected from 89 elective adenoidectomy or adenotonsillectomy patients at Satakunta Central Hospital in Pori, Finland, between April 2008 and March 2009. Inclusion criteria were adenoidectomy or adenotonsillectomy according to clinical indication and written informed consent from the study subject or his/her guardian.^1^ The study protocols were approved by the Ethics Committee of Satakunta Central Hospital and the Ethics Committee of the Hospital District of Southwest Finland.

### 2.2 Sample collection

The internal part of the tonsil and/or adenoid tissue removed from the patient was instantly cut into 3–4 mm cubes and stored in RNAlater (Qiagen), first at +2–8°C until the next working day, and then at −80°C.^1^ NPA samples were collected through a nostril using a standardized procedure.^17^ If the aspirate yield was small, the collection was repeated after administering 2 ml of physiologic saline into a nostril. For viral analyses, a piece of the removed tonsils and/or adenoid and the NPA were stored at −80°C. Study patients completed a standard questionnaire to collect information on health, medication, and respiratory symptoms within 30 days before the operation.^1^

### 2.3 Study outcomes

The primary outcome of this study was to analyze the presence of different human herpesviruses and respiratory viruses in adenoid tissues. The secondary outcome was to compare common herpes‐ and respiratory virus findings between adenoid, tonsil, and NPA samples.

### 2.4 Virus diagnostics

Viruses in adenoid tissue, tonsil tissue, and NPA were detected by polymerase chain reactions (PCRs; including the reverse transcription step when applicable) on nucleic acid extracts.^17^ Commercial tests were performed according to manufacturer's instructions. GeneProof PCR kits (GeneProof) were used for cytomegalovirus (CMV) and EBV. Laboratory design real‐time quantitative PCRs were used for herpes simplex virus 1 (HSV1), herpes simplex virus 2 (HSV2), varicella‐zoster virus (VZV), HHV6, and HHV7.^18^, ^19^

For respiratory virus detection in all sample types, laboratory design real‐time PCRs were used for HBoV1, EV, RV, and RSV, as described earlier.^20^, ^21^, ^22^ In adenoid tissue samples, Allplex respiratory panels I–III (Seegene) were used for multiplex PCR detection of adenovirus (AdV); human bocavirus 1 to 4 (HBoV); coronavirus (CoV) 229E, NL63, and OC43; enterovirus (EV); influenza A and B viruses (FluA and FluB); metapneumovirus (MPV); parainfluenza virus (PIV) types 1 to 4; respiratory syncytial virus (RSV); and rhinovirus (RV). In tonsil tissue and NPA samples, the Seeplex RV12 ACE Detection (Seegene) multiplex PCR kit was used to detect respiratory viruses including AdV, CoV (229E/NL63 and OC43/HKU1), Flu A and Flu B, MPV, PIV types 1–3, RSV group A and B, and RV. PCR tests were done at the Department of Virology, University of Turku, Finland.

### 2.5 Statistical analysis

Data were analyzed using SPSS software Statistics for Windows, version 27.0 (IBM SPSS Statistics for Macintosh). Categorical variables are expressed as frequencies and percentages and were analyzed by using a Chi‐square test or Fisher's exact test (when counts <5). Continuous variables are described as medians and were analyzed using the Mann–Whitney *U* test due to skewed distribution. Cohen's Kappa was used to determine agreement between most common intratonsillar, intra‐adenoid, and NPA viruses. Agreement statistics between results from PCR tests from intra‐adenoid, intratonsillar, and NPA virus diagnostics were calculated with kappa coefficients and could be interpreted as follows: <0.40 = poor, ≥0.40 to <0.75 = fair to good, and ≥0.75 = excellent.^23^ Virus DNA levels were tested for equality between adenoids and tonsils by using the Wilcoxon signed‐rank test. Statistical significance was established at the level of two‐sided *p* < 0.05.

## 3 RESULTS

### 3.1 Study population

Initially, 200 adeno‐ and/or tonsillectomy patients were consecutively recruited year‐round. Of these study patients, 45 received adenoidectomies and 44 had adenotonsillectomies, and they were included. Sole tonsillectomy patients (111) were excluded from this study since their tonsil samples have been utilized and studied earlier.^1^ Finally, 89 study patients had sufficient samples and were included in this study. Forty‐five autologous adenoid and NPA samples were analyzed and 44 autologous adenoid, NPA, and tonsil samples, respectively.

### 3.2 Patient characteristics

The median age of the study patients (*n* = 89) was 5 years (range: 1–20); 62% were male. The most common indication for the operation was adenoid and/or tonsillar hypertrophy in 43 (48%). There were mixed indications (otitis media with effusion, tonsillar/adenoid hypertrophy, recurrent tonsillitis) in 33 (37%) cases. Mild upper respiratory tract symptoms such as rhinitis, cough, throat pain, otalgia, and upper respiratory tract obstruction during the operation day were reported by 24/77 (31%) of the study patients (Supporting Information: Table S1).

### 3.3 Total virus findings

Ninety‐seven percent (86/89) of the adenoid tissue samples were positive for ≥1 viruses. At least one intratonsillar virus was detected in 95% (42/44) of adenotonsillectomy patients. Moreover, one or more viruses were detected in 94% (84/89) of the NPA samples (Table 1). One or more different herpesviruses were found in 89% of the samples and one or more different respiratory viruses in 94% of the samples.

**Table 1:** Detected viruses in nasopharyngeal aspirate, intra‐adenoid, and intratonsillar samples.

Virus	Intra‐adenoid (*n* = 89)		Intratonsillar (*n* = 44)		Nasopharyngeal (*n* = 89)	
	With or without other virus	Sole	With or without other virus	Sole	With or without other virus	Sole
Human herpesvirus 6	29 (33%)	1 (1%)	20 (45%)	5 (11%)	41 (46%)	0
Human herpesvirus 7	23 (26%)	1 (1%)	23 (52%)	6 (14%)	34 (38%)	1 (1%)
Epstein–Barr virus	22 (25%)	2 (2%)	10 (23%)	0	22 (25%)	1 (1%)
Cytomegalovirus	1 (1%)	0	0	0	13 (15%)	0
Herpes simplex virus 1	1 (1%)	0	0	0	2 (2%)	0
Herpes simplex virus 2	0	0	0	0	0	0
Varicella‐zoster virus	0	0	0	0	0	0
Rhinovirus	53 (60%)	4 (4%)	3 (7%)	0	56 (63%)	9 (10%)
Human bocavirus	52 (58%)	3 (3%)	10 (23%)	1 (2%)	31 (35%)	1 (1%)
Adenovirus	11 (12%)	1 (1%)	8 (18%)	0	23 (26%)	1 (1%)
Enterovirus	6 (7%)	0	7 (16%)	2 (5%)	9 (10%)	0
Coronavirus^a^	1 (1%)	1 (1%)	0	0	8 (9%)	1 (1%)
Parainfluenza virus types 1–4	12 (13%)	2 (2%)	3 (7%)	0	6 (7%)	0
Influenza A or B virus	1 (1%)	0	0	0	4 (4%)	1 (1%)
Respiratory syncytial virus	8 (9%)	0	2 (5%)	0	2 (2%)	0
Metapneumovirus	0	0	1 (2%)	0	0	0
No virus findings	3 (3%)		2 (5%)		5 (6%)	
≥1 viruses	86 (97%)		42 (95%)		84 (94%)	

*Note*: Values are expressed as number of positive findings (%) *n* = 89 or *n* = 44.

^a^ Coronavirus types 229E, NL63, OC43, or HKU1.

### 3.4 Herpesvirus findings

**Figure 1:**
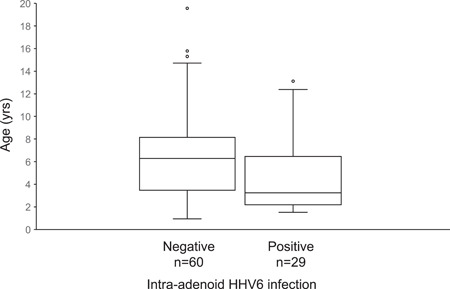
Age distributions of human herpesvirus 6 (HHV6) positive and negative patients. Patients with intra‐adenoid HHV6‐positive findings were younger (median age 3) compared to patients with negative polymerase chain reaction test (median age 6).

HHV6 was the most prevalent herpesvirus (33%) of the adenoid samples, followed by HHV7 (26%) and EBV (25%). Only one adenoid sample was positive for HSV1. VZV and HSV2 were not detected (Table 1). The detection rates of herpesviruses in tonsils were HHV6, 45%; HHV7, 52%; and EBV, 23%. Other herpesviruses were not found in tonsils. The DNA levels (copies/mm^3^) of HHV6 and HHV7 were significantly higher in tonsil tissue compared to adenoid tissue (*p*‐values 0.002 and <0.001, respectively). EBV genome levels were not significantly different in adenoid and tonsil tissues. In one case, the EBV virus DNA load was 210 000 copies/mm^3^ in tonsil tissue, which could be considered as a marker for acute infectious mononucleosis or reactivation of the virus (Table 2). The herpesvirus detection rates were slightly higher in NPA samples compared with adenoid tissue: HHV6, 46%; HHV7, 38%; and EBV, 25%. Exceptionally, the CMV PCR test was positive in 15% of the NPA samples and in only one (1%) of the adenoid samples. HHV7 was the most common virus found concurrently in all three sample types (9/44, 20%) (Table 3). Patients with intra‐adenoid HHV6‐positive findings were younger compared to those having a negative PCR test (median age 3 years vs. 6 years, respectively, *p* = 0.017) (Figure 1). Otherwise, no associations between age or gender and virus findings in adenoid or tonsils samples were found.

**Table 2:** DNA levels of human herpesvirus 6 (HHV6), human herpesvirus 7 (HHV7), and Epstein–Barr virus (EBV) in adenoid and tonsil tissue samples of adenotonsillectomy patients (Wilcoxon signed rank test).

Virus	Virus DNA copies/mm^3^ tissue, mean (range), in adenoid tissue (*n* = 44)	Virus DNA copies/mm^3^ tissue, mean (range), in tonsil tissue (*n* = 44)	*p*‐value
HHV6	5.5 (0–62.5)	81.9 (0–1137)	0.002
HHV7	12.3 (0–79.1)	95.3 (0–1107)	<0.001
EBV	37.5 (0–873.6)	4807.6 (0–205 071)	0.12

**Table 3:** Site of the virus findings.

Virus	Any site (*n* = 89)	Adenoid only (*n* = 89)	Tonsil only (*n* = 44)	NPA only (*n* = 89)	Tonsil^a^ and adenoid (*n* = 44)	Adenoid and NPA (*n* = 89)	Tonsil^a^ and NPA (*n* = 44)	All sites (*n* = 44)
Human herpesvirus 6	60 (67%)	7 (8%)	7 (16%)	19 (21%)	5 (11%)	14 (16%)	5 (11%)	3 (7%)
Human herpesvirus 7	45 (50%)	3 (3%)	4 (9%)	12 (13%)	4 (9%)	7 (8%)	6 (14%)	9 (20%)
Epstein–Barr virus	31 (35%)	5 (6%)	2 (5%)	6 (7%)	2 (5%)	10 (11%)	1 (2%)	5 (11%)
Cytomegalovirus	13 (15%)	0	0	12 (13%)	0	1 (1%)	0	0
Herpes simplex virus 1	2 (2%)	0	0	1 (1%)	0	1 (1%)	0	0
Herpes simplex virus 2	0	0	0	0	0	0	0	0
Varicella‐zoster virus	0	0	0	0	0	0	0	0
Rhinovirus	70 (79%)	14 (16%)	0	16 (18%)	0	37 (42%)	1 (2%)	2 (5%)
Human bocavirus	58 (65%)	22 (25%)	2 (5%)	4 (4%)	3 (7%)	22 (25%)	0	5 (11%)
Adenovirus	31 (35%)	2 (2%)	3 (7%)	16 (18%)	3 (7%)	5 (6%)	1 (2%)	1 (2%)
Enterovirus	14 (16%)	0	3 (7%)	4 (4%)	2 (5%)	3 (3%)	1 (2%)	1 (2%)
Coronavirus^b^	8 (9%)	0	0	7 (8%)	0	1 (1%)	0	0
Parainfluenza virus types 1–4	17 (19%)	8 (9%)	3 (7%)	2 (2%)	0	4 (4%)	0	0
Influenza A or B	4 (4%)	0	0	3 (3%)	0	1 (1%)	0	0
Respiratory syncytial virus	11 (12%)	7 (8%)	2 (5%)	1 (1%)	0	1 (1%)	0	0
Metapneumovirus	1 (1%)	0	1 (2%)	0	0	0	0	0

^a^ Tonsil samples from adenotonsillectomy patients, *n* = 44.

^b^ Coronaviruses 229E, OC43, NL63, and HKU1.

### 3.5 Respiratory virus findings

RV (60%) was the most prevalent respiratory virus in adenoid tissue samples followed by HBoV1 (58%). Other respiratory viruses detected in adenoid tissue were parainfluenza viruses 1–4 (13%), adenovirus (12%), and others (<10% each). In tonsils, HBoV1 (23%) and adenovirus (18%) were the two most prevalent respiratory viruses. Moreover, in NPA samples RV (63%) was the most common respiratory virus (Table 1). In 36% of the adenoid samples, two different viruses were detected, the most common combination being RV and HBoV1 (8%). Concerning three different sample types, HBoV1 was the most common virus found only in adenoid tissues (25%) (Table 3). Patients with intra‐adenoid and intratonsillar HBoV1 were younger than those without (4 years vs. 8 years, *p* = 0.001 and *p* = <0.001). Additionally, patients with a positive adenovirus finding in intra‐adenoid tissue were younger than those having a negative PCR test (3 years vs. 6 years, *p* = 0.029). An HBoV1‐negative result was more often associated with male gender than female gender (*p* = 0.019). No other associations between age or gender and virus findings in adenoid or tonsil tissue were found.

### 3.6 Agreement between sample sites

HHV7 was frequently detected both in adenoid and tonsil tissue of the same patient (kappa value 0.55, 95% confidence interval: 0.34–0.77). Similarly, detection of EBV in adenoids agreed strongly with detection of the virus in the corresponding tonsils (kappa value 0.67, 95% confidence interval: 0.39–0.94). Furthermore, EBV detection showed agreement between adenoid and NPA samples (kappa value 0.58) and between NPA and tonsil samples (kappa value 0.53). Of the two most commonly detected respiratory viruses, HBoV1 and RV, HBoV1 was more frequently detected in adenoids than in tonsils (kappa value 0.3) or NPAs (kappa value 0.38). RV was more frequently detected in adenoids and NPA than in tonsils (Table 4). There was no significant agreement between the detection of RV in adenoids and corresponding NPAs (kappa values 0.27) or tonsils (kappa value 0.024).

**Table 4:** Comparison of adenoid versus tonsil, adenoid versus NPA, and NPA versus tonsil virus detections.

	Adenoid versus tonsil (*n* = 44)				Adenoid versus NPA (*n* = 89)				NPA versus tonsil (*n* = 44)			
Virus	Adenoid PCR	Tonsil PCR		Kappa coefficient (95% CI)	Adenoid PCR	NPA PCR		Kappa coefficient (95% CI)	NPA PCR	Tonsil PCR		Kappa coefficient (95% CI)
		neg	pos			neg	pos			neg	pos	
HHV6	neg	20 (83%)	12 (60%)	‐	neg	36 (75%)	24 (58%)	‐	neg	13 (54%)	12 (60%)	‐
	pos	4 (17%)	8 (40%)	0.24 (−0.029, 0.51)	pos	12 (25%)	17 (42%)	0.17 (−0.03, 0.37)	pos	11 (46%)	8 (40%)	−0.059 (−0.35, 0.23)
HHV7	neg	21 (100%)	10 (43%)	‐	neg	48 (87%)	18 (53%)	‐	neg	15 (71%)	8 (35%)	‐
	pos	0 (0%)	13 (57%)	**0.55 (0.34, 0.77)**	pos	7 (13%)	16 (47%)	0.37 (0.17, 0.56)	pos	6 (29%)	15 (65%)	0.37 (0.091, 0.64)
EBV	neg	32 (94%)	3 (30%)	‐	neg	60 (90%)	7 (32%)	‐	neg	31 (91%)	4 (40%)	‐
	pos	2 (6%)	7 (70%)	**0.67 (0.39, 0.94)**	pos	7 (10%)	15 (68%)	**0.58 (0.38, 0.77)**	pos	3 (9%)	6 (60%)	**0.53 (0.23, 0.83)**
RV	neg	18 (44%)	1 (33%)	‐	neg	19 (58%)	17 (30%)	‐	neg	14 (34%)	0 (0%)	‐
	pos	23 (56%)	2 (67%)	0.024 (−0.11, 0.15)	pos	14 (42%)	39 (70%)	0.27 (0.063, 0.47)	pos	27 (66%)	3 (100%)	0.066 (−0.012, 0.14)
HBoV1	neg	21 (63%)	2 (20%)	‐	neg	33 (57%)	4 (13%)	‐	neg	23 (67%)	5 (50%)	‐
	pos	13 (37%)	8 (80%)	0.301 (0.056, 0.55)	pos	25 (43%)	27 (87%)	0.38 (0.21, 0.55)	pos	11 (33%)	5 (50%)	0.146 (−0.14, 0.44)

*Note*: Values are expressed as *n* (%). Bold values indicates Kappa coefficient ≥ 0.40 to < 0.75 (means fair to good agreement).

Abbreviations: CI, confidence interval; EBV, Epstein–Barr virus; HBoV1, human bocavirus 1; HHV6, human herpesvirus 6; HHV7, human herpesvirus 7; NPA, nasopharyngeal aspirate; PCR, polymerase chain reaction; RV, rhinovirus

## 4 DISCUSSION

To our knowledge, this is the first study to cover multiplex detection of herpesvirus (HHV1‐2, VZV, EBV, CMV, HHV6–7) and respiratory viruses in adenoid, tonsil, and NPA samples. Our study shows a few essential findings of prevalent virus infections in adenoid and tonsils of nonacutely ill adenotonsillar surgery patients. First, overall herpesvirus prevalence was high. HHV6, HHV7, and EBV were commonly found in adenoids and tonsils. Additionally, there were observed agreements in the detection of HHV7 in adenoids and corresponding tonsils and EBV in all sample types. Second, higher herpesvirus genome loads were found in tonsils compared to adenoids. Only detection of HHV6 was associated with age. Third, RV and HBoV1 were the most frequently detected respiratory viruses overall. For neither virus were the detections in corresponding samples from different sites connected.

At least one virus was found in 97% of the adenoid samples, 95% of the tonsil samples, and 94% of the NPA samples. In an earlier study concerning only respiratory viruses, the detection rates in the same sample types were 86%, 69%, and 79%, respectively.^5^ Moreover, in another study (median age 6 years) the detection rate of at least one herpesvirus (EBV, CMV, HHV6, HHV7, HHV8) in adenoid and tonsil tissue was 100%.^8^ Our results are in line with earlier ones and demonstrate the abundance of viruses in Waldeyer's ring lymphatic organs in chronic adenotonsillar disease patients.

HHV6 was the most prevalent (33%) intra‐adenoid herpesvirus, and the prevalence was similar compared to earlier studies.^8^, ^10^ Conversely, the intratonsillar prevalence of HHV6 (46%) was higher than in our earlier study (16%) but similar to the SPLIT study (51%).^4^, ^7^ The younger age of HHV6‐positive patients is logical considering that HHV6 is an etiologic agent of childhood exanthema subitum (roseola infantum), which usually presents in children between 6 and 12 months of age.^24^ Furthermore, adenoid tissue is most active in children under 6 years old.^25^ Our finding contrasts Comar et al., who reported that HHV6‐positivity in the adenoid tissue was higher in the >5 years old group compared to <5 years.^10^ In adults HHV6 has been linked to autoimmune diseases.^26^, ^27^ Moreover, HHV6 can cause severe infections such as hepatitis and encephalitis in immunosuppressed patients.^10^, ^27^

HHV7 was the most common virus detected in tonsils. HHV6 and HHV7 virus DNA levels were significantly higher in tonsil tissues compared to adenoid tissues. Our study agrees with earlier results concerning HHV7, but contradicts those of HHV6.^8^ The interplay between HHV6 and HHV7 has been suggested in immunosuppressive and immunocompetent children. Primary infection of one virus may trigger the other virus to reactivate.^28^ HHV7 may also cause childhood exanthema subitum, but the clinical presentation is more obscure. Fever and seizures have been associated with viremic HHV7 infection in small children.^28^, ^29^ Our results support earlier studies showing that herpesviruses persist in adenoid and tonsil tissues after primary infection.

In the present study, the prevalence of EBV was similar in all corresponding samples compared. Similar results have been seen in an earlier study comparing EBV DNA load between adenoids and tonsils.^30^ Interestingly, 25% of the NPA samples were EBV‐positive. EBV persists in B cells and EBV genome found from NPA samples is most obviously originated from lymphocytes present in the mucus and harboring the virus. EBV has been the most common herpesvirus found from adenoid tissue in children, the prevalence being 40%–80%.^8^, ^9^, ^16^ In a study comparing children and adults, EBV prevalence was slightly higher in the adenoid tissue of adults (74%) compared to children (66%).^30^

It is known that 90% of people are infected by EBV. In Finland and Western countries, 50%–60% of primary EBV infections occur in childhood without symptoms or with mild symptoms. In adolescents and young adults EBV can cause acute mononucleosis. Typical symptoms and findings include fever, sore throat, and swollen and exudative tonsils.^14^, ^31^ The role of EBV in the pathogenesis of various diseases has been exclusively studied. EBV is strongly associated with Burkitt's lymphoma, Hodgkin's lymphoma, and NK/T‐cell lymphoma.^14^, ^15^ In the endemic area of southern China and South‐East Asia, 95%–98% of nasopharyngeal carcinomas are associated with EBV.^32^, ^33^ Whereas in Finland, where the incidence is low, a nationwide study showed that 62% of the nasopharyngeal carcinoma tumor samples were EBV‐positive.^34^

In our study, CMV was detected in NPA but not in tonsils and only in one adenoid sample. Our results are in line with the SPLIT study, but another study has detected CMV in adenoids and tonsils.^7^, ^8^ CMV infections persist lifelong in the host and cause severe morbidity in neonates and immunocompromised patients.^12^ Moreover, CMV infects a variety of cells, for example, endothelial cells and dendritic cells of tonsils.^35^ In our study, CMV was basically not found in secondary lymphoid tissue. Therefore, the virus finding in NPA may be a result of virus shedding.

The prevalence of common respiratory viruses in adenoid, tonsils, and NPA is well known.^1^, ^5^, ^6^, ^36^, ^37^ In our study, RV was the most prevalent respiratory virus in adenoid and NPA samples (60% and 63%, respectively), but the prevalence in tonsils was only 7%. This is in line with earlier studies using PCR, which show that RV is common in adenoid tissue but not in tonsil tissue.^5^, ^38^ In our study, RV was not found in tonsils without concurrent detection of the virus in NPA. Interestingly, no RV was detected in NPA samples of 14 patients with RV in adenoid tissue. This may be due to faster resolution of RV from nasopharynx than from adenoids after infection.

HBoV1 was the most prevalent respiratory virus in tonsil tissue (23%), and the frequency of HBoV1 was also high in adenoid tissue and NPA samples. Detection of HBoV1 or RV did not show agreement between sample types, which is in line with earlier results.^5^ Furthermore, 40% of the HBoV1‐positive cases were detected only in adenoid tissue samples. This confirms earlier results, that HBoV1 is more likely to be found in adenoid tissue than in tonsils or NPA.^5^ It has been shown that adenoid tissue is an important site for HBoV1 persistence in children,^5^, ^6^ and even high loads of HBoV1 DNA load can be found in tonsils without acute infection.^3^ Moreover, HBoV1 infection is shown to have immunomodulatory effects.^39^, ^40^, ^41^ The immunomodulatory effects of viruses on local lymphoid tissue level have been studied,^1^, ^2^ but the differences between immune responses in adenoid and tonsil tissue are not completely known. Tonsillectomy, but not adenoidectomy, as an effective treatment to periodic fever, aphthous stomatitis, pharyngitis, and adenitis (PFAPA) syndrome suggests that these two secondary lymphoid tissue compartments differ in immune functions.^42^

The strength of this study is the characterization of common herpesviruses and respiratory viruses in three different sample types obtained from the same patient. The present study shows that many different viruses are prevalent in secondary lymphoid tissue in chronic adenotonsillar patients. Adenoid and tonsil tissues serve as reservoirs for multiple herpes‐ and respiratory viruses. Since certain viruses have the potential to modulate immune functions and associate with carcinogenesis, our results offer interesting knowledge of virus prevalence in active secondary lymphoid tissues.

The limitations of the study include that we did not study healthy control patients for ethical reasons. Additionally, the respiratory virus tests from tonsils and NPA were done earlier than those from adenoids using a test kit version, which was later replaced with a new test kit by the manufacturer. Virus infections were not studied at the cellular level.

## 5 CONCLUSION

This study shows high overall virus prevalence and characterizes the wide spectrum of different herpesviruses and common respiratory viruses in adenoid and tonsil tissues of nonacutely ill adenotonsillar surgery patients. Furthermore, it shows the differences in herpesvirus prevalence between adenoid, tonsils, and NPA sample types. Adenoid and tonsil tissues may serve as virus reservoirs in nonacutely ill patients and warrant future studies.

## AUTHOR CONTRIBUTIONS

The study protocol and manuscript were written by the investigators. Data were collected by Tuomo Puhakka, Lotta E. Ivaska, and Emilia Mikola and analyzed by Lotta E. Ivaska, Antti Silvoniemi, and Tuomas Jartti. Viral analyses were carried out by Matti Waris and Tytti Vuorinen. The study was supervised by Tuomas Jartti. The first draft was written by Lotta E. Ivaska. All authors contributed to the revision of the final manuscript.

## CONFLICTS OF INTEREST

The authors declare no conflicts of interest.

## Supporting information

Supporting information.

## Data Availability

The data that support the findings of this study are available from the corresponding author upon reasonable request.
