# Supplementary material for: Herpesvirus infections in adenoids in patients with chronic adenotonsillar disease
Source: J Med Virol. 2022 May 11;94(9):4470–7. doi: 10.1002/jmv.27818 (PMC9545566; doi:10.1002/jmv.27818)
Supplement: Supplementary file 1 — Supporting information. [file JMV-94-4470-s001.docx]

**Table 1.** Patient characteristics of adenoidectomy and adenotonsillectomy patients

| **Factor** | **n=89** |
| --- | --- |
| Median age (range), years | 5 (1–20) |
| Male | 55 (62%) |
| Adenotomy  Adenotonsillectomy | 45 (51%)  44 (49%) |
| Indication for adenotomy/adenotonsillectomy |  |
| Recurrent otitis/otitis media with effusion | 9 (10%) |
| Recurrent tonsillitis | 2 (2%) |
| Adenoid/tonsil hypertrophy/obstructive sleep apnea | 43 (48%) |
| Mixed indications | 33 (37%) |
| Periodic fever | 2 (2%) |
| Self-reported allergy | 34/80 (43%) |
| Physician-diagnosed asthma | 11/78 (14%) |
| Self-reported allergic rhinitis | 16/75 (21%) |
| Physician-diagnosed atopic dermatitis | 14/80 (18%) |
| Smoking or exposure to smoking | 43/83 (52%) |
| Mild respiratory symptoms on the operation day* | 24/77 (31%) |
| Respiratory symptoms 2 weeks prior to the operation | 37/73 (51%) |

* Rhinitis, cough, throat pain, otalgia, upper respiratory tract obstruction
